# Anxiety and depression among patients with chronic obstructive pulmonary disease and general population in rural Nepal

**DOI:** 10.1186/s12888-017-1550-5

**Published:** 2017-12-12

**Authors:** Niresh Thapa, Muna Maharjan, Tirtha Man Shrestha, Srijana Gauchan, Prakash Pun, Yam Bahadur Thapa

**Affiliations:** 1 Department of Gynecological Oncology, Second Clinical College of Wuhan University, Zhongnan Hospital, Wuhan, Hubei China; 2 Department of General Practice and Emergency Medicine, Karnali Academy of Health Sciences, Jumla, Nepal; 3 Second Clinical College of Wuhan University, Hope School of Nursing, Wuhan, Hubei China; 4 Department of General Practice and Emergency Medicine, Maharajgunj Medical Campus, Tribhuwan University Teaching Hospital, Kathmandu, Nepal; 5 Department of General Practice and Emergency Medicine, Patan Academy of Health Sciences, Lalitpur, Nepal; 6 Department of Medicine, United Mission Hospital, Palpa, Nepal; 7 Department of General Practice, Okhaldhunga Community Hospital, Okhaldhunga, Nepal

**Keywords:** Chronic obstructive pulmonary disease, Anxiety, Depression

## Abstract

**Background:**

Anxiety and depression are usually under diagnosed among the patients with Chronic Obstructive Pulmonary Disease (COPD), which has a negative impact on patient quality of life through restriction of activities, loss of independence, and decreased social functioning. The purpose of this study was to describe the levels and characteristics of anxiety and depression in patients with COPD in Nepal as compared to the general population.

**Methods:**

A hospital-based observational comparative analytical study was conducted in the United Mission Hospital, Tansen and the Okhaldhunga Community Hospital, Okhaldhunga, Nepal from June 1st 2015 to April 15th 2016. A convenience sample of two groups of participants were recruited: patients with COPD (study group) and visitors to the facility (comparison group). Anxiety and depression were measured with the Beck Anxiety and Depression Inventory Scale.

**Results:**

A total of 198 individuals participated in the study; 93 with COPD and 105 from the general population. The mean age of the respondents was 58.24 ± 12.04 (40-82) years. The mean scores for anxiety and depression in COPD group were 23.76± 9.51 and 27.72± 9.37 respectively, while in comparison group, the mean score for anxiety was 8.01± 6.83 and depression was 11.60 ± 8.42. Both anxiety and depression scores were statistically significant between the groups with *p* value <0.001.

**Conclusions:**

Anxiety and depression were almost three times more common in COPD patients compared to the participants from the general population. Early assessment and multi-model treatment of anxiety and depression should be part of management in COPD.

## Background

COPD is characterized by usually slowly progressive and persistent air flow limitation condition which is a common preventable and treatable disease. Tobacco smoking, noxious particles or gases are often associated with an enhanced chronic inflammatory response in the airways and the lung (Global Initiative for Chronic Obstructive Lung Disease, GOLD) [1]. More than 16 million persons in the United States are affected by COPD and it is the fourth leading cause of death in world, which will rise to the third most common cause of death worldwide by 2020. It is one of the important public health issue [2, 3].

There is a high prevalence of COPD with an estimated 1.5 to 2 million asthmatics out of total 26 million populations in Nepal. Many patients in Nepal with COPD are illiterate and come from a poor economic status [2, 4, 5].

Health-related quality of life may have a major impact on COPD patients. They may have restriction of activities, decreased social functioning and loss of independence [6]. **C**OPD has multiple co-morbidities. Anxiety and depression are two of the most common and least-treated co-morbidities among COPD patients [7, 8]. However, only a few prospective studies have addressed how to diagnose and manage these disorders and determine their impact on health status among patients with COPD[9].

A study conducted in Nepal by Risal et al., reported, “crude prevalence of anxiety was 22.7 % and of depression 11.7%. The age and gender –adjusted prevalence of hospital anxiety and depression scale (HADS) for anxiety was 16.1% and for depression 4.2%” [10].

COPD associated death rate has doubled in the past 30 years, which indicates the healthcare system’s failure to address this problem. Increased mortality, decreased functional status, and decreased quality of life in these patients have been linked to psychiatric co-morbidities, particularly anxiety and depression [11].

Few studies were found worldwide on COPD associated with psychiatric co-morbidities and far less in Nepal. Depression and anxiety have been found to negatively affect COPD treatment, including pulmonary rehabilitation. This study compared the levels and characteristics of anxiety and depression in patients with COPD and the general population in rural Nepal.

## Methods

An observational comparative analytical cohort study was conducted in the United Mission Hospital, Tansen and Okhaldhunga Community Hospital, Okhaldhunga, from June 1st 2015 to April 15th 2016. A total of 198 respondents, study group (*N* = 93) and comparison group (*N* = 105), who gave consent were included in the study. The inclusion criteria of respondents for study group were patients age above 40 years, with post bronchodilator, ratio of forced expiratory volume in 1 s and forced vital capacity (FEV1/FVC) <70% and Beta2-agonist reversibility <15% (GOLD criteria for the diagnosis of COPD) [1], history of smoking, admitted COPD patients and for comparison group patient visitor visiting in United Mission Hospital, Palpa and Okhaldhunga Community Hospital, Okhaldhunga. The exclusion criteria included unwilling to participate in the study, patient having other co-morbidities which included known psychiatric disorders patient, and other significant respiratory or inflammatory disease: asthma, pulmonary tuberculosis, congestive cardiac failure, interstitial lung disease and taking medicines like Beta- blockers and antipsychotics.

### Data collection

Permission was taken from The General Practice and Emergency Medicine departments, Institute of Medicine and respective institutions prior to the data collection. The participants were recruited from patients admitted with COPD (study group) and apparently healthy people basically patients’ visitors (comparison group) in the United Mission Hospital (105 respondents; COPD/Comparison- 46/59) and Okhaldhunga Community Hospital (93 respondents; COPD/Comparison- 47/46). Those clients willing to give consent were included in the study and the surveys were administered at that time. For those clients who could not read or write, questions were asked verbally and filled by the trained person or researcher.

### Measurements

The Beck Anxiety Inventory (BAI) and the Beck Depression Inventory (BDI) questionnaire were used to assess the anxiety and depression level of respondents. The BAI was used to measure the severity of anxiety consisting of 21 multiple-choice questions. Each question has graded from 0 to 3 with a score of 3 indicating the greatest severity. BAI has score ranging from 0 to 63. The total score of 0-7 indicates minimal anxiety, 8-15 mild anxiety, 16-25 moderate anxiety, and 26-63 severe anxiety. The BDI also consists of 21 multiple-choices questions graded from 0 to 3, with a score of 3 indicating the greatest severity of depression. BDI has a scores ranging from 0 to 63. The total score of 0-13 represents healthy, 14-19 minimal level of depression, 20-28 mild depression, and 29-63 severe depression [12–14].

### Statistical analysis

The data were entered, coded and analyzed by using the Statistical Package for Social Sciences (SPSS) version 16. Baseline demographics and clinical characteristics were compared among the groups using cross tabulation and chi square tests. *P* < 0.05 was regarded as statistical significance.

## Results

### Sample

The total number of respondents was 198 (COPD/comparison group: 93/105). The demographic and clinical characteristics are summarized in Table 1 with the mean age of 58.01 + 12.11 (40-82) years. A majority (66.9%) of the respondents in COPD group had a history of smoking tobacco whereas, in comparison group 41.9% had a history of smoking tobacco.

**Table 1:** Socio-demographic and clinical characteristics of respondents

Characteristics	Study population (*N* = 198)	COPD group (*N* = 93)	Control group (*N* = 105)
Age (mean age ± SD) (Min-Max)	58.01 ± 12.11 (40-82)	64.93 ± 9.19 (40-82)	51.86 ± 11.06 (40-76)
Sex			
Male	119 (60.1%)	41 (44.1%)	78 (74.3%)
Female	79 (39.9%)	52 (55.9%)	27 (25.7%)
Occupation			
Farmer	107 (54%)	47 (50.5%)	60 (57.1%)
Home maker	46 (23.2%)	31 (33.3%)	15 (14.3%)
Retired from service	22 (11.1%)	12 (12.9%)	10 (9.5%)
Service holder	12 (6.1%)	0 (0%)	12 (11.4%)
Labor	9 (4.5%)	3 (3.2%)	6 (5.7%)
Business	2 (1.0%)	0 (0%)	2 (1.9%)
Marital status			
Married	166 (83.8%)	74 (79.6%)	92 (87.6%)
Widow	24 (12.1%)	19 (20.5%)	5 (4.8%)
Separated	5 (2.5%)	0 (0%)	5 (4.8%)
Single	3 (1.5%)	0 (0%)	3 (2.9%)
Family type			
Joint	158 (79.8%)	80 (86.0%)	78 (74.3%)
Nuclear	40 (20.2%)	13 (14.0%)	27 (25.7%)
Education			
Illiterate	120 (60.6%)	76 (81.7%)	44 (41.9%)
Informal education	24 (12.1%)	4 (4.3%)	20 (19.0%)
Primary level	32 (16.2%)	10 (10.8%)	22 (21.0%)
Secondary level	22 (11.1%)	3 (3.2%)	19 (18.1%)
Smoking status			
Smoker	133 (67.2%)	89 (95.7%)	44 (41.9%)
Non smoker	65 (32.8%)	4 (4.3%)	61 (58.1%)
Alcohol consumption	77 (38.9%)	29 (31.2%)	48 (45.7%)
Disease duration (mean year ± SD)		6.75 ± 5.89	
Hospitalization in last one year		1.88 ± 0.99	
BAI, mean ± SD (range 0 - 45)*	15.41 ± 11.35	23.76 ± 9.51	8.01 ± 6.83
BDI, mean ± SD (range 1 - 48)*	19.18 ± 11.97	27.72 ± 9.37	11.60 ± 8.42

*Chi square test value: *P* value <0.05

### Anxiety

The mean score of anxiety in the COPD group was 23.76 ± 9.51 and in the comparison group, it was 8.01 ± 6.83. Among the COPD group, 37 (39.8%) had a severe anxiety level, meanwhile in comparison group only 3 (2.9%) had a severe anxiety level (Table 2).

**Table 2:** Beck Anxiety Inventory Score Level among the Respondents

Group	Anxiety Scale					*P* value* (χ^2^)
	Minimal anxiety	Mild anxiety	Moderate anxiety	Severe anxiety	Total	
COPD	4 (4.3%)	13 (14.0%)	39 (41.9%)	37 (39.8%)	93 (100%)	<0.001
Control	59 (56.2%)	32 (30.5%)	11 (10.5%)	3 (2.9%)	105 (100%)	
Total	63 (31.8%)	45 (22.7%)	50 (25.3%)	40 (20.2%)	198 (100%)	

*Chi square value

### Depression

The mean score for depression in the COPD group was 27.72 ± 9.37 and for the comparison group, the mean score for depression was 11.60 ± 8.42. As with the scores for depression, 34 (35.5%) of COPD group respondents had severe depression whereas it was only 5 (4.8%) among comparison group (Table 3).

**Table 3:** Beck depression inventory score level among the respondents

Group	Depression scale					*P* value* (χ^2^)
	Minimal depression	Mild depression	Moderate depression	Severe depression	Total	
COPD	4 (4.3%)	14 (15.1%)	41 (44.1%)	34 (36.6%)	93 (100%)	<0.001
Control	71 (67.6%)	23 (21.9%)	6 (5.7%)	5 (4.8%)	105 (100%)	
Total	75 (37.9%)	37 (18.7%)	47 (23.7%)	39 (19.7%)	198 (100%)	

* Chi square value

### Characteristics of anxiety and depression

Gender, occupation, and number of hospitalization were not correlated with scores of BDI and BAI for both groups. However, the age and level of anxiety were statistically significant within the COPD group (*p* = 0.03) and the comparison group (*p* < 0.001). Similarly, age showed significant association with the level of depression in the COPD group (*p* < 0.05).

Family type and level of anxiety had a significant association with *p* = 0.02 (COPD group) and *p* = 0.001 (comparison group) (Tables 4 and 5). Education level of the respondents showed significant association with the level of depression with p = 0.001(Table 5).

**Table 4:** Association between Beck Anxiety Inventory Score Level and different variables of Respondents

Group/Variables	Anxiety scale					*P* value* (χ^2^)
	Minimal anxiety	Mild anxiety	Moderate anxiety	Severe anxiety	Total	
COPD Group						0.03
Less than 60 years	0 (0%)	2 (8.0%)	7 (28%)	16 (64.0%)	25 (100%)	
Above 60 years	4 (5.9%)	11 (16.2%)	32 (47.1%)	21 (20.9%)	68 (100%)	
Control Group						0.00
Less than 60 years	45 (55.6%)	30 (37.0%)	6 (7.4%)	0 (0%)	81 (100%)	
Above 60 years	14 (58.3%)	2 (8.3%)	5 (20.8%)	3 (12.5%)	24 (100%)	
COPD Group						0.8
Male	2 (4.9%)	7 (17.1%)	17 (41.5%)	15 (36.6%)	41 (100%)	
Female	2 (3.8%)	6 (11.5%)	22 (42.3%)	22 (42.3%)	52 (100%)	
Control Group						0.1
Male	42 (53.8%)	27 (34.6%)	6 (7.7%)	3 (3.8%)	78 (100%)	
Female	17 (63.0%)	5 (18.5%)	5 (18.5%)	0 (0%)	27 (100%)	
COPD Group						0.02
Nuclear family	0 (0%)	5 (38.5%)	6 (46.2%)	2 (15.4%)	13 (100%)	
Joint family	4 (5.0%)	8 (10.0%)	33 (41.2%)	35 (43.8%)	80 (100%)	
Control Group						0.001
Nuclear family	24 (88.9%)	3 (11.1%)	0 (0%)	0 (0%)	27 (100%)	
Joint family	35 (44.9%)	29 (37.2%)	11 (14.1%)	3 (3.8%)	78 (100%)	
COPD Group						0.32
Illiterate	2 (2.6%)	10 (13.2%)	32 (42.1%)	32 (42.1%)	76 (100%)	
Literate	2 (11.8%)	3 (17.6%)	7 (41.2%)	5 (29.4%)	17 (100%)	
Control Group						0.01
Illiterate	20 (45.5%)	13 (29.5%)	8 (18.2%)	3 (6.8%)	44 (100%)	
Literate	39 (63.9%)	19 (31.1%)	3 (4.9%)	0 (0%)	61 (100%)	
COPD Group						0.1
Smoker	4 (4.5%)	11 (12.4%)	39 (43.8%)	35 (39.3%)	89 (100%)	
Non smoker	0 (0%)	2 (50.0%)	0 (0%)	2 (50.0%)	4 (100%)	
Control Group						0.01
Smoker	23 (52.3%)	10 (22.7%)	8 (18.2%)	3 (6.8%)	44 (100%)	
Non smoker	36 (59.0%)	22 (36.1%)	3 (4.9%)	0 (0%)	61 (100%)	

*Chi-square test was used

**Table 5:** Association between beck depression inventory score level and different variables of respondents

Group/Variables	Depression scale					*P* value* (χ^2^)
	Minimal depression	Mild depression	Moderate depression	Severe depression	Total	
COPD Group						0.009
Less than 60 years	0 (0%)	5 (20.0%)	5 (20.0%)	15 (60.0%)	25 (100%)	
Above 60 years	4 (5.9%)	9 (13.2%)	36 (52.9%)	19 (27.9%)	68 (100%)	
Control Group						0.3
Less than 60 years	56 (69.1%)	16 (19.8%)	6 (7.4%)	3 (3.7%)	81 (100%)	
Above 60 years	15 (62.5%)	7 (29.2%)	0 (0%)	2 (8.3%)	24 (100%)	
COPD Group						0.9
Male	2 (4.9%)	7 (17.1%)	18 (43.9%)	14 (34.1%)	41 (100%)	
Female	2 (3.8%)	7 (13.5%)	23 (44.2%)	20 (38.5%)	52 (100%)	
Control Group						0.06
Male	56 (71.8%)	13 (16.7%)	6 (7.7%)	3 (3.8%)	78 (100%)	
Female	15 (55.6%)	10 (37.0%)	0 (0%)	2 (7.4%)	27 (100%)	
COPD Group						0.7
Nuclear family	0 (0%)	3 (23.1%)	5 (38.5%)	5 (38.5%)	13 (100%)	
Joint family	4 (5.0%)	11 (13.8%)	36 (45.0%)	29 (36.2%)	80 (100%)	
Control Group						0.1
Nuclear family	18 (66.7%)	6 (22.2%)	0 (0%)	3 (11.1%)	27 (100%)	
Joint family	53 (67.9%)	17 (21.8%)	6 (7.7%)	2 (2.6%)	78 (100%)	
COPD Group						0.34
Illiterate	2 (2.6%)	12 (15.8%)	35 (46.1%)	27 (35.5%)	76 (100%)	
Literate	2 (11.8%)	3 (11.8%)	6 (35.3%)	7 (41.2%)	17 (100%)	
Control Group						0.001
Illiterate	25 (56.8%)	14 (31.8%)	0 (0%)	5 (11.4%)	44 (100%)	
Literate	46 (75.4%)	9 (14.8%)	6 (9.8%)	0 (0%)	61 (100%)	
COPD Group						0.06
Smoker	4 (4.5%)	14 (15.7%)	41 (46.1%)	30 (33.7%)	89 (100%)	
Non smoker	0 (0%)	0 (0%)	0 (0%)	4 (100%)	4 (100%)	
Control Group						0.001
Smoker	21 (47.7%)	18 (40.9%)	3 (6.8%)	2 (4.5%)	44 (100%)	
Non smoker	50 (82.0%)	5 (8.2%)	3 (4.9%)	3 (4.9%)	61 (100%)	

*Chi-square test was used

## Discussion

This study aimed to describe the levels of anxiety and depression among COPD patients in comparison to the general population. The study demonstrated that majority of the COPD group reported moderate to severe anxiety and depression in comparison to the general population. Chronic somatic diseases are often associated with psychiatric co-morbidities [15]. There is a high prevalence rate of depression and anxiety among patients with asthma and COPD. Higher prevalence of anxiety and depression are observed among patients with COPD even in a mild stage than the general population [16]. It is estimated that anxiety and depression are six times more prevalent in asthmatics in comparison to the general population however some controversies are there. [17].

A similar study reported that anxiety and depression differed by age group: clinically significant levels of depression and anxiety were more prevalent in patients aged less than 60 years, irrespective of clinical severity of COPD [6]. This result contradicts the present study findings that more respondents with age more than 60 years have moderate to severe level of anxiety and depression than those with age less than 60 years.

The risk factors for increased rates of anxiety and depression include gender, lack of support and having more severe diseases in COPD. In comparison to gender, females have higher rate of anxiety and depression, and the rates of depression are more strongly correlated with severity of the disease than males [16]. Similarly, Di Marcto et al. reported that female gender had a high prevalence of anxiety and depression [18]. In the present study higher number females score moderate to severe in both anxiety and depression scale than males, however there was no statistical significant relation between the scores of anxiety/depression and respondent’s socio-demographic and clinical characteristics.

Kunik et al. reported that anxiety and depression in COPD patients in a general practitioner’s outpatient clinic were 19% and 23% respectively which is very much consistent with the present study findings [19]. These high rates stress the importance of screening and treatment of depression and anxiety in patients with COPD to maintain health and functional status, prevent unnecessary hospitalizations, dropout from pulmonary rehabilitation or poor outcomes from smoking cessation programs.

Treatment for COPD is now aimed at immediately relieving and reducing the impact of symptoms, as well as reducing the risk of future adverse events such as exacerbations. A framework for multi-modal COPD management that matches individualized assessment of the disease to these treatment objectives will better meet each patient’s needs [1].

Rates of anxiety and depression are high in COPD patients as it is a chronic condition, which needs regular follow up and treatment. In addition to the increased risk of COPD due to tobacco smoking, people living in remote areas use firewood to cook food and their homes are not well ventilated which causes indoor air pollution and increased risk for development of COPD and recurrent exacerbation. These recurrent attacks leads to increased number of readmission in hospital. Due to the effects of COPD on overall health, most patients are unable to work, may be a huge burden for family members, and may be abandoned.

Pooler reported that managing depression and anxiety starts with an accurate diagnosis. To improve the patient’s condition, identification of those who have more permanent and sustained anxiety and depression is necessary, along with developing screening methods and implementing effective management strategies to alleviate the impact of the co-morbidities [20].

COPD with anxiety and depression affects family and society as well as the individual patient. Thus it should be addressed accordingly. Medical as well as psychological management is important in the treatment of COPD, counseling to the patient and family about the smoking cessation, proper clothing, immunization, regular follow up and use of medicine.

## Conclusion

In this study, the BAI and BDI scores for anxiety and depression were significantly higher in patients with COPD compared to a sample from the general population. Assessment and management of anxiety and depression are important as these common co-morbidities can negatively impact treatment compliance, make it difficult to control COPD, and lead to increased morbidity and mortality. Future studies should focus on evaluating the effectiveness of multi-modal approaches to management of anxiety and depression in COPD. Examples include the effectiveness of different types of pharmacologic treatment, cognitive behavior therapy and the collaborative-care model for pulmonary rehabilitation.

## Abbreviations

BAI: Beck Anxiety Inventory
BDI: Beck Depression Inventory
COPD: Chronic obstructive pulmonary disease
FEV: Forced Expiratory Volume
FVC: Forced Vital Capacity
GOLD: Global Initiative for Chronic Obstructive Pulmonary Disease
